# Association of Lesion Location With Long-Term Recovery in Post-stroke Aphasia and Language Deficits

**DOI:** 10.3389/fneur.2019.00776

**Published:** 2019-07-24

**Authors:** Bomi Sul, Kyoung Bo Lee, Bo Young Hong, Joon Sung Kim, Jaewon Kim, Woo Seop Hwang, Seong Hoon Lim

**Affiliations:** Department of Rehabilitation Medicine, St. Vincent's Hospital, College of Medicine, The Catholic University of Korea, Seoul, South Korea

**Keywords:** stroke, aphasia, K-WAB, prognosis, outcome, brain imaging, VLSM

## Abstract

**Background:** Recovery from post-stroke aphasia is important for performing the activities of daily life, returning to work, and quality of life. We investigated the association between specific brain lesions and the long-term outcome of four dimensions of aphasia: fluency, comprehension, naming, and repetition 12 months after onset in patients with stroke.

**Methods:** Our retrospective cross-sectional observational study investigated the relationship between the Korean version of the Western Aphasia Battery scores in 31 stroke patients 1 year after the onset of stroke and stroke lesion location. Brain lesions were assessed using voxel-based lesion symptom mapping (VLSM) in conjunction with magnetic resonance imaging.

**Results:** Damage to the Rolandic cortex, Heschl's gyrus, the posterior corona radiata, supramarginal cortex, superior longitudinal fasciculus, superior temporal gyrus, and insula was associated with a low total AQ score. Lesions in the inferior triangularis and inferior operculum of the frontal cortex, supramarginal cortex, and insula were associated with a poor fluency outcome. Damage to the parietal cortex, angular cortex, temporal middle cortex, sagittal stratum, and temporal superior cortex was associated with poor recovery of comprehension skills. Lesions in the angular cortex, supramarginal cortex, posterior corona radiata, superior longitudinal fasciculus, internal capsule, temporal superior cortex, and temporal middle cortex were associated with poor recovery of naming in patients with stroke. Damage to the superior temporal cortex, posterior corona radiata, and superior longitudinal fasciculus was associated with poor recovery of repetition component.

**Conclusions:** We identified specific brain lesions associated with long-term outcomes in four dimensions of aphasia, in patients with post-stroke aphasia. Our findings may be useful for advancing understanding for the pathophysiology of aphasia in stroke patients.

## Introduction

Aphasia is a language disorder typically caused by stroke-related damage to the dominant hemisphere. Post-stroke aphasia is associated with mortality, disability, and the use of health services. The long-term outcomes for post-stroke aphasia vary widely. Recovery from aphasia is important for performing the activities of daily life, returning to work, and quality of life in patients with stroke (1).

Several studies have investigated the prognosis of speech and language after stroke. Factors predicting post-stroke aphasia recovery include lesion size and location, aphasia severity, and the type of speech deficit (2). Additionally, stroke severity at onset and the Western Aphasia Battery (WAB) score 72 h after onset (3, 4), and the specific brain regions damaged, affect the long-term prognosis of post-stroke aphasia (2, 5–8). The involvement of Broca's area, the superior temporal gyrus, arcuate fasciculus, and superior longitudinal fasciculus are associated with a poor prognosis in patients with chronic post-stroke aphasia at 4–6 months after onset (5, 6, 8). However, the timing of 4–6 months after onset might be less sufficient to reflect full functional recovery, further neurological recovery is thought to be likely (9). Knowledge of the role or specific brain lesions may facilitate the understanding of the pathophysiology of aphasia and could then be used as basis for neuromodulation strategies for aphasia, such as repetitive trans-magnetic stimulation or trans-cranial electric stimulation (10–12).

Thus, we investigated the roles that specific brain lesions play in recovery of deficits in fluency, comprehension, naming and repetition of language, using lesion symptom mapping methods that included analyses of brain magnetic resonance imaging (MRI) scans and clinical language test, in patients with first-ever supratentorial strokes 12 months after stroke onset.

## Methods

### Study Design and Participants

Our retrospective observational study included the data of 31 post-stroke aphasia, right-handed patients with first-stroke patients recruited from a single inpatient/outpatient center between January 2011 and December 2017. The inclusion criteria were: (1) first-ever supratentorial stroke, (2) a single ischemic or hemorrhagic left hemisphere lesion confirmed by magnetic resonance imaging (MRI), (3) right handedness, (4) native Korean speaker, (5) at least 9-years of education, (6) no prior history of psychiatric or neurological disorders, (7) absence of central or peripheral paretic dysarthria, and (8) completion of a language assessment using the Korean version of the Western Aphasia Battery (K-WAB) 1 year after the onset of stroke (6). No restrictions were placed on the type or severity of the aphasia. Subjects were excluded if they had any other functional or structural brain disorder. Similarly, to previous studies for brain lesion analysis, we included the patients with stroke, regardless of type; ischemic and hemorrhagic (13–15). Of the 214 individuals who underwent language assessment for aphasia after stroke, 31 qualified for the study. All subjects received speech therapy and other rehabilitation therapy; physical or occupational therapies as needed. The rehabilitation program of all subjects had started within 5 days after onset. The speech therapy continued up to 12 months after onset, and consisted of with 0.5–2 h per week, respectively.

Demographic characteristics and language assessments were obtained for all subjects. High-resolution 1.5T anatomical MRI scans with 5-mm slice thickness were used to determine the precise location of the brain lesion (16). All participants underwent the same MRI scanning protocol. We used a 1.5-Tesla Philips MR scanner (ACHIEVA, Philips, Netherlands). The MRI protocol included whole-brain, three-dimensional, T1-weight images (TR/TE = 527.4/13, Slice thickness 5 mm, GAP 1.5 mm, flip angle 90', Refocus angle 180', FOV 230^*^230, Matrix 304^*^222, Voxel size 0.75^*^1.02^*^5 mm, NEX 2), T2-weight images (TR/TE = 4500/100, Slice thickness 5 mm, GAP 1.5 mm, flip angle 90', Refocus angle 160', FOV 230^*^230, Matrix 384^*^233, Voxel size 0.6^*^0.86^*^5 mm, NEX 2), and fluid-attenuated inversion-recovery (FLAIR) axial images (TR/TE = 6000/100, Slice thickness 5 mm, GAP 1.5 mm, flip angle 90', Refocus angle 150', FOV 230^*^230, Matrix 304^*^222, Voxel size 0.75^*^1.02^*^5 mm, NEX 2). The study protocol was reviewed and approved by the Institutional Review Board of The Catholic University, College of Medicine (Registry No. VC18RESI0112). The requirement for informed consent was waived by the board.

### Language Test

The validated K-WAB was administered to all patients on admission by a single speech language pathologist; however, only data from cases of aphasia caused by first-ever stroke were subjected to analysis. The K-WAB assessment consists of four subsets: fluency, comprehension, repetition, and naming (7, 17). The severity of aphasia was quantified using the aphasia quotient (AQ; range, 0–100), which was calculated using the formula developed by Kertesz (fluency score + comprehension score/20 + naming score/10 + repetition score/10) ×2 (17). The data of K-WAB measured at 1 year after onset were used for outcome of language recovery.

### Lesion Analysis and Statistical Analysis

Lesion locations and sizes were assessed using MRIcron software (http://www.mricro.com/mricron). T2 images were co-registered with each participant's T1 MRI, and then, the T1 and lesion maps were normalized to the Montreal Neurologic Institute (MNI) template using statistical parametric mapping 8 software (SPM8, http://www.fil.ion.ucl.ac.uk/spm/software/spm8) and non-parametric mapping (NPM) software (18–20). The number of MRI voxels in each stroke lesion was calculated, and the lesions were traced by a trained image analyst and confirmed by an experienced physiatrist (a neurorehabilitation specialist), who was blind to all clinical data. Only voxels indicating that at least 10% (*n* = 3) of the patients had lesions were included in the final analysis. The non-parametric Brunner–Munzel test for continuous data was used (15). Colored VLSM maps representing the *z* statistics were generated and overlaid onto the automated anatomical labeling and Johns Hopkins University white matter templates provided with the MRIcron software (18, 21).

## Results

The study included 31 patients (mean age, 55.5 ± 11.5 years; 15 females and 16 males). The mean time from the onset of stroke to the language assessment was 725.9 ± 233.4 days, and the mean brain lesion volume was 55482.16 ± 43109.30 voxels. Patient clinical and demographic data are shown in Table 1. The language assessment findings are shown in Supplementary Table 1.

**Table 1:** Patient demographic and clinical characteristics.

**Demographics (*n* = 31)**	
Gender, M/F (%)	51.6/48.4
Age, years^*^	55.5 ± 11.5
Time from onset of stroke to speech evaluation, days^*^	725.9 ± 233.4
Stroke pathology, hemorrhage/infarction (%)	51.6/48.4
Brain injury location (*n*, %)	
Cortex	14 (45.2)
Subcortex	6 (19.3)
Mixed (cortex and subcortex)	11 (35.5)
Lesion Volume voxels (*n*)^*^	55482.16 ± 43109.30

* *Mean ± SD. M, male; F, female*.

**Figure 1:**
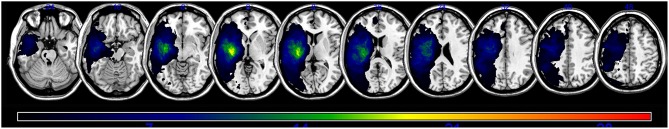
A lesion overlap map of all subjects (*n* = 31). The color spectrum indicates the frequency of overlap.

**Figure 2:**
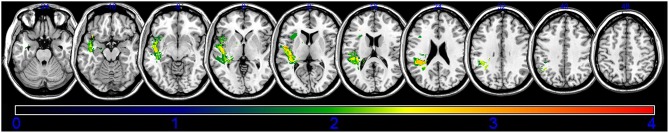
Voxel-based lesion-symptom mapping of the total aphasia quotient after application of the non-parametric Brunner–Munzel test. The color scale indicates Brunner–Munzel rank order *z* statistics. Only voxels significant at *p* < 0.05 are shown. The statistical map shows voxels with a minimum *Z* score of 1.89797 and maximum range of 4, which was the maximum brightness.

**Figure 3:**
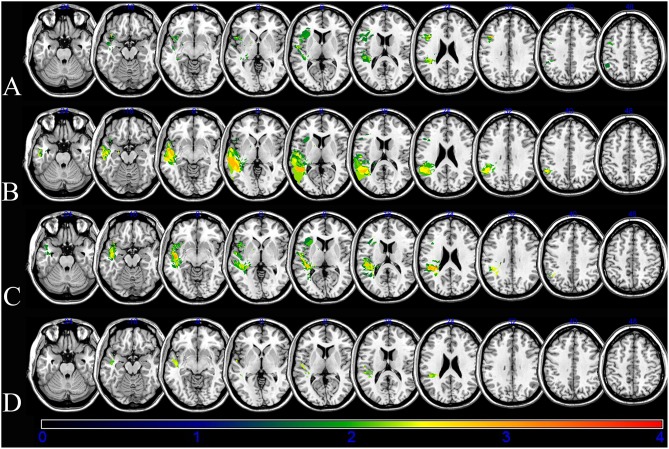
Voxel-based lesion-symptom mapping (VLSM) of the language deficits after the non-parametric Brunner–Munzel statistical analysis. The color scale indicates Brunner–Munzel rank order z statistics. Only voxels significant at *p* < 0.05 are shown. The maximum range of the *z* score was set at 4, which was the maximum brightness. **(A)** VLSM for fluency with a minimum *z* score of 2.32076. **(B)** VLSM for comprehension with a minimum *Z* score of 2.50055. **(C)** VLSM for naming with a minimum *Z* score of 2.38888. **(D)** VLSM for repetition with a minimum *Z* score of 3.19465.

An overlap map of the 31 lesions was created (Figure 1). The VLSM analyses using NPM revealed that lesions of the Rolandic cortex, Heschl's gyrus, posterior corona radiata, supramarginal cortex, superior longitudinal fasciculus (SLF), superior temporal gyrus, and insula were associated with a low total AQ (Table 2, Figure 2). The frontal inferior triangularis, frontal inferior operculum, supramarginal cortex, and insula were associated with fluency; the parietal cortex, angular cortex, temporal middle cortex, sagittal stratum, and temporal superior cortex were associated with comprehension; the angular cortex, supramarginal cortex, posterior corona radiata, SLF, internal capsule, temporal superior cortex, and temporal middle cortex were associated with naming; and the temporal superior cortex, posterior corona radiata, and SLF were associated with repetition (Table 3, Figure 3).

**Table 2:** Total aphasia quotients associated with stroke lesions.

**MNI coordinates** **(X, Y, Z)**	**BM *Z* max**	***n* Voxels**	**Anatomical brain lesion**
−49, −5, 8	2.71638	101	Rolandic cortex
−38, −25, 8	2.9998	110	Heschl
−29, −37, 24	3.03567	111	Posterior corona radiata
−46, −35, 28	2.94784	114	Supramarginal cortex
−37, −39, 25	2.77033	111	Superior longitudinal fasciculus
−45, 31, 16	2.5758	87	Temporal superior
−39, −15, 12	1.89797	85	Insula

*Montreal Neurological Institute (MNI) coordinates of voxels using lesion overlay map of 31 subjects that were significant based on the Brunner–Munzel (BM) Z score and the number (n) of clustering voxels that survived the false discovery rate-corrected threshold of P < 0.05. Anatomical regions were identified using the automated anatomical labeling and Johns Hopkins University white matter templates*.

**Table 3:** Stroke lesions associated with the aphasia dimensions.

**Sub-quotients**	**MNI coordinates (X, Y, Z)**	**BM *Z* max**	***n* Voxels**	**Patients with lesion (*n*)**	**Anatomical brain lesion**
Fluency	−36, 15, 31	3.23888	83	11	Frontal inferior triangularis
	−37, 14, 30	3.23888	85	11	Frontal inferior operculum
	−43, −35, 26	2.32076	113	7	Supramarginal cortex
	−35, 12, 2	2.48052	98	12	Insula
Comprehension	−47, −46, 40	2.50055	113	6	Parietal cortex
	−47, −48, 35	2.50055	113	6	Angular cortex
	−44, −48, 6	2.88614	107	5	Temporal middle cortex
	−43, −29, −6	2.90267	114	11	Sagittal stratum
	−54, −4, −10	3.35279	107	7	Temporal superior cortex
Naming	−42, −55, 39	2.38888	102	5	Angular cortex
	−44, −37, 28	2.43480	116	5	Supramarginal cortex
	−28, −37, 23	3.15591	108	5	Posterior corona radiata
	−38, −40, 25	2.77023	111	6	Superior longitudinal fasciculus
	−36, −37, 13	2.78821	109	10	Internal capsule
	−45,−12,−9	3.10543	116	12	Temporal superior cortex
	−44, −1, −17	2.55562	110	7	Temporal middle cortex
Repetition	−46, −10, −10	3.61530	117	12	Temporal superior cortex
	−30, −38, 23	3.61530	110	5	Posterior corona radiata
	−37, −37, 26	3.19465	111	6	Superior longitudinal fasciculus

*Montreal Neurological Institute (MNI) coordinates of voxels using lesion overlay map of 31 subjects that were significant based that were significant based on the Brunner–Munzel (BM) Z score and the number (n) of clustering voxels that survived the false discovery rate-corrected threshold of p < 0.05. Anatomical regions were identified using the automated anatomical labeling and Johns Hopkins University white matter templates*.

The damage of supramarginal cortex affected fluency and naming. The temporal middle cortex and angular cortex related with recovery of comprehension and naming. The SLF and posterior corona radiata may affect the recovery of repetition and naming. The damage of the temporal superior cortex may affect outcome of comprehension, naming and repetition.

## Discussion

We found that lesion location was associated with long-term outcomes in fluency, comprehension, naming, and repetition in patients with aphasia at 12 months after stroke onset. Damage to the Rolandic cortex, Heschl's gyrus, posterior corona radiata, supramarginal cortex, superior longitudinal fasciculus, superior temporal gyrus, and insula were associated with overall poor outcomes. Lesions in the inferior triangularis and inferior operculum of the frontal cortex, supramarginal cortex, and insula were associated with poor fluency. Damage to the parietal cortex, angular cortex, temporal middle cortex, sagittal stratum, and temporal superior cortex was associated with poor comprehension skills. Lesions in the angular cortex, supramarginal cortex, posterior corona radiata, superior longitudinal fasciculus, internal capsule, temporal superior cortex, and temporal middle cortex were associated with poor recovery of naming ability, and damage to the superior temporal cortex, posterior corona radiata, and superior longitudinal fasciculus was related to poor recovery of repetition skills. These findings suggest that specific lesion sites are predictors of aphasia recovery in patients with first-ever stroke 12 months after onset.

Nowadays, neuromodulation with repetitive magnetic stimulation or trans-cranial electric stimulation for stroke patients has been widely investigated (12, 22–25). For neuromodulation, determining target specific brain lesion is important (10, 24, 26). Thus, the functions of specific brain lesions for stroke have been uncovered; motor of upper limb, gait, balance, and language (6, 13, 19, 27). These investigations would be useful for understanding of functional deficit of stroke, itself, and also for determining target brain lesion in neuromodulation therapy. Our findings may contribute to the understanding of aphasia itself, and further to usage of neuromodulation therapy of aphasia.

Previous studies have shown that the language network involving Broca's complex, including the inferior prefrontal gyrus, insular cortex, Wernicke's complex, premotor cortex, and superior temporal gyrus play a major role in language (6, 28, 29). Moreover, white matter including the arcuate fascicle, SLF, uncinate fascicle, and the extreme capsule fiber system play a functional role in language processing (5, 7, 29). Another recent study showed that the damage to the arcuate fasciculus related to the recovery of aphasia, and damage to the external capsule also affected the recovery of aphasia (14). Our finding that lesions in the supramarginal cortex, superior temporal gyrus, insula, and SLF were associated with a low AQ score in patients with stroke is consistent with that of previous studies. A previous case report wrote that the Rolandic cortex is the cause of transcortical motor aphasia (30), the Heschl's gyrus is involved in the comprehension of syntax (31), and posterior corona radiata lesions are associated with poor recovery from aphasia (32). These lesions had not been considered as the main cause for aphasia (8). Our findings suggest that the Rolandic cortex, Heschl's gyrus, and posterior corona radiata may be new causative lesions for post-stroke aphasia. Taken together, these findings suggest that the Rolandic cortex, Heschl's gyrus, and posterior corona radiata play roles in the recovery of post-stroke aphasia via the lesion itself, or in disruptions of language network (7).

The role of the Broca's complex in verbal fluency is well-known (28); however, our findings suggest that the inferior triangularis and inferior operculum also play significant roles in mediating fluency. Moreover, the insula cortex has been shown to support fluency as a syntax-specific process (33). A recent lesion-symptom correlational analysis found that the posterior supramarginal gyrus played a role in the processing of concrete and abstract verbs related to fluency (34). We found that damage to the inferior triangularis and inferior operculum of the frontal cortex, supramarginal cortex, and insula were associated with poor fluency. Language fluency is a complex process involving phonation, motor function, syntax synthesis, and semantic meaning; thus, it is not surprising that it is mediated by a network of brain regions. The recent repetitive transcranial magnetic stimulation was consistent with the inhibition of contra-lesional pars triangularis and pars opecularis (24, 35, 36). Our results support the rationale of the target for neuromodulation.

The results that parietal cortex, angular cortex, temporal middle cortex, and temporal superior cortex lesions were associated with poor recovery of comprehension is consistent with previous findings (37–39). Moreover, our finding that the sagittal stratum pathway may mediate language comprehension supports that of a previous study showing that sagittal stratum damage, including the geniculostriate pathway and inferior longitudinal fasciculus, impaired access to orthographic word forms and semantics (40). A recent study of post-stroke aphasia found that the posterior superior temporal gyrus, intraparietal sulcus, and pars triangularis were involved in naming ability (41). We found that the superior temporal cortex, temporal middle cortex, angular cortex, supramarginal cortex, posterior corona radiata, superior longitudinal fasciculus, and internal capsule were associated with poor recovery of naming in stroke patients with aphasia. Naming is higher-order function involving comprehension, semantic, syntax, phonation, and speech. Thus, several lesion sites may affect naming ability.

We found that damages to the superior temporal cortex, posterior corona radiata, and SLF were associated with poor repetition outcomes in post-stroke aphasia patients. A previous study found that the left inferior fronto-occipital fascicle and uncinate fascicle were associated with repetition (42). Another study, which had a small sample (11 aphasic subjects), found that impaired repetition was associated with lesions in the central operculum, angular gyrus, supramarginal gyrus, and Heschl's gyrus in the acute phase of stroke (43). Taken together, these findings indicate that the brain regions involved in repetition deficits were related to those in fluency and comprehension and the white matter connecting them.

The supramarginal cortex, temporal superior and middle cortex, angular cortex, SLF, and posterior corona radiata contributed to two or more functions. Possible explanations were as follows. First, these areas would contribute to several roles, indirectly. Thus, influence from a distinct area might affect our results. Based on the brain network, the studies for a specific brain lesion may be not sufficient to explain all pathogenesis of aphasia. Second, four subsets of aphasia have developed, based on clinical phenotype. The mismatch between the process in brain and clinical phenotype would contribute to our results. However, the diagnosis and treatment should be reliant on clinical phenotype. Our results revealed the specific brain lesion on long-term outcome of aphasia with four subsets; fluency, comprehension, repetition, and naming, in patients with stroke. These results would be useful in the clinical setting. For example, for the planning of non-invasive neuromodulation therapy, our results may have merit for the decision of the target area individually, based on the patient's MRI findings and K-WAB results.

Our study has two major limitations. First, our small sample size and cross-sectional design may limit the interpretation of our findings. Second, our sample may have been affected by selection bias because we excluded the data of subjects who died 12 months post-stroke; thus, the study included relatively well patients who would not normally undergo the K-WAB test 12 months post-stroke. Nonetheless, we identified specific brain areas associated with four language deficits in patients with first-ever stroke 12 months after onset. Our study differs from those conducted previously in that we investigated lesion locations associated with four aphasia deficits using VLSM, and we used data obtained 12 months after stroke onset when further neurological recovery is thought to be unlikely (6, 42, 43).

In conclusion, we identified specific brain lesions associated with long-term outcome with K-WAB and acute MRI data in four language deficits 12 months after onset in stroke patients with aphasia using VLSM. Our findings may be useful for the development of treatment strategies and for advancing understanding of the pathophysiology of aphasia in stroke patients.

## Data Availability

All datasets generated for this study are included in the manuscript and/or the Supplementary Files.

## Ethics Statement

The study protocol was reviewed and approved by the Institutional Review Board of The Catholic University, College of Medicine (Registry No. VC18RESI0112). The requirement for informed consent was waived by the board.

## Author Contributions

BS and KL: making concept, analysis of results, and writing draft. JSK and BH: providing subjects and review of draft. JK: analysis of results and acquisition of data. WH: acquisition of data. SL: making concept, analysis of results, writing draft, review, and finalize of draft.

### Conflict of Interest Statement

The authors declare that the research was conducted in the absence of any commercial or financial relationships that could be construed as a potential conflict of interest.
