# Supplementary material for: Association of Lesion Location With Long-Term Recovery in Post-stroke Aphasia and Language Deficits
Source: Front Neurol. 2019 Jul 24;10:776. doi: 10.3389/fneur.2019.00776 (PMC6668327; doi:10.3389/fneur.2019.00776)
Supplement: Supplementary file 1 [file Table_1.DOCX]

Supplementary Table 1. Results of the language assessment (n=31)

|  | Aphasia quotient | Fluency | Comprehension | Naming | Repetition |
| --- | --- | --- | --- | --- | --- |
| 1 | 34.1 | 11 | 45 | 13 | 25 |
| 2 | 29.7 | 17 | 194 | 98 | 100 |
| 3 | 48.8 | 7 | 134 | 63 | 44 |
| 4 | 55.6 | 8 | 147 | 65 | 60 |
| 5 | 40.3 | 5 | 115 | 44 | 50 |
| 6 | 32.5 | 5 | 97 | 41 | 23 |
| 7 | 0 | 0 | 0 | 0 | 0 |
| 8 | 90.6 | 18 | 172 | 91 | 96 |
| 9 | 97.4 | 19 | 200 | 97 | 100 |
| 10 | 20.2 | 4 | 116 | 3 | 0 |
| 11 | 74.9 | 12 | 171 | 79 | 90 |
| 12 | 45.5 | 4 | 135 | 58 | 62 |
| 13 | 80.5 | 16 | 157 | 72 | 92 |
| 14 | 2.4 | 0 | 24 | 0 | 0 |
| 15 | 28.1 | 8 | 93 | 4 | 10 |
| 16 | 21.3 | 8 | 51 | 1 | 0 |
| 17 | 21.6 | 4 | 76 | 3 | 27 |
| 18 | 26.4 | 6 | 112 | 0 | 16 |
| 19 | 55.3 | 11 | 135 | 61 | 38 |
| 20 | 53.4 | 16 | 0 | 65 | 12 |
| 21 | 22 | 7 | 16 | 15 | 17 |
| 22 | 52.2 | 3 | 126 | 51 | 57 |
| 23 | 0 | 0 | 0 | 0 | 0 |
| 24 | 16.7 | 4 | 31 | 18 | 10 |
| 25 | 6 | 1 | 40 | 0 | 0 |
| 26 | 86.4 | 17 | 176 | 84 | 90 |
| 27 | 31.9 | 5 | 117 | 21 | 30 |
| 28 | 12.3 | 3 | 63 | 0 | 0 |
| 29 | 88.6 | 14 | 184 | 87 | 94 |
| 30 | 21.3 | 6 | 77 | 3 | 5 |
| 31 | 78.5 | 16 | 145 | 70 | 90 |

K-WAB = Korean version of the Western Aphasia Battery.

Maximum scores: aphasia quotient out of 100; fluency, 20; comprehension, 200; naming, 100; and repetition, 100.
